# Boron neutron capture therapy induces cell cycle arrest and cell apoptosis of glioma stem/progenitor cells in vitro

**DOI:** 10.1186/1748-717X-8-195

**Published:** 2013-08-06

**Authors:** Ting Sun, Zizhu Zhang, Bin Li, Guilin Chen, Xueshun Xie, Yongxin Wei, Jie Wu, Youxin Zhou, Ziwei Du

**Affiliations:** 1 Neurosurgery & Brain and Nerve Research Laboratory, The First Affiliated Hospital of Soochow University, Suzhou, Jiangsu, China; 2 Beijing Capture Tech Co., Ltd, Beijing, China

**Keywords:** Boron neutron capture therapy, Glioma stem/progenitor cells, Radiosensitivity

## Abstract

**Background:**

Glioma stem cells in the quiescent state are resistant to clinical radiation therapy. An almost inevitable glioma recurrence is due to the persistence of these cells. The high linear energy transfer associated with boron neutron capture therapy (BNCT) could kill quiescent and proliferative cells.

**Methods:**

The present study aimed to evaluate the effects of BNCT on glioma stem/progenitor cells in vitro. The damage induced by BNCT was assessed using cell cycle progression, apoptotic cell ratio and apoptosis-associated proteins expression.

**Results:**

The surviving fraction and cell viability of glioma stem/progenitor cells were decreased compared with differentiated glioma cells using the same boronophenylalanine pretreatment and the same dose of neutron flux. BNCT induced cell cycle arrest in the G2/M phase and cell apoptosis via the mitochondrial pathway, with changes in the expression of associated proteins.

**Conclusions:**

Glioma stem/progenitor cells, which are resistant to current clinical radiotherapy, could be effectively killed by BNCT in vitro via cell cycle arrest and apoptosis using a prolonged neutron irradiation, although radiosensitivity of glioma stem/progenitor cells was decreased compared with differentiated glioma cells when using the same dose of thermal neutron exposure and boronophenylalanine pretreatment. Thus, BNCT could offer an appreciable therapeutic advantage to prevent tumor recurrence, and may become a promising treatment in recurrent glioma.

## Introduction

Human glioblastoma multiforme (GBM) is the most aggressive type of brain tumors and has a poor prognosis. Although current therapies (based on surgical resection, radiotherapy and chemotherapy) modestly improve patient survival, about 75% of patients will die within 2 years of diagnosis [1]. There is currently no cure for GBM, because the surgeon cannot completely remove this diffuse tumor. Radiotherapy remains the key component of GBM treatment, and most attempts to increase its intensity were hampered by unacceptable late toxicities [2]. There are increasing evidences that GBM possess small numbers of ‘stem-like’ cells, called glioma stem cells (GSC), which are able to give rise to a new tumor closely similar to the original cancer. The almost inevitable GBM recurrence is due to the persistence of these cells despite multimodality treatment [3,4]. It has been proposed that these tumor-initiating cells are resistant to radiation therapy, and that this property contributes to the poor treatment outcomes associated with these tumors [5,6]. Since escalating treatment intensity is associated with unacceptable damage to normal brain, alternative methods of overcoming this resistance are urgently required.

Boron neutron capture therapy (BNCT) theoretically provides a way to selectively destroy boron-10 (^10^B)-loaded malignant cells, sparing normal cells without ^10^B. As stated by Barth and his co-authors [7], BNCT is based on the nuclear capture and fission reactions that occur when ^10^B, a nonradioactive boron isotope, is irradiated using low-energy thermal neutrons. This fission reaction produce high linear energy transfer (LET) α particles (^4^He) and lithium-7 (^7^Li) nuclei.

For BNCT to be successful, the tumor must contain high levels of ^10^B (~20 μg/g or ~10^9^ atoms/cell), while peripheral normal tissue must contain only small levels. In addition, a sufficient amount of thermal neutrons must be delivered to the tumor tissue to sustain a lethal ^10^B(n,a)^7^Li reaction [8,9]. The destructive effects of these high-energy particles, which only have a limited penetration power in tissue (5–9 μm), are thus limited to boron-containing cells [7-9]. The high LET radiation associated with BNCT would kill anoxic and quiescent cells, as well as oxygenated and proliferative cells [10]. In theory, GSC would be killed by neutrons as long as a sufficient dose of ^10^B is absorbed by the GCS.

Radiosensitivity of tumor cells is associated with cell differentiation [11]. The characteristics of glioma stem/progenitor cells (GSPC) SU2, which were used in this research, were between glioma stem cells and progenitor cells. The SU2 cells are radioresistant to certain doses of X-rays [12]. To the best of our knowledge, there is a lack of relevant data on the effects of BNCT on GSPC. The purpose of the present study was to assess the in vitro effects of BNCT on GSPC and differentiated glioma cells, using the In-Hospital Neutron Irradiator (IHNI-1), which is a low-power research reactor using a miniature neutron source [13].

## Materials and methods

### Cell lines

The SU2 cell line, generously provided by Professor Qiang Huang, is a GSPC line [14,15]. The SU2 cells were cultured in serum-free DMEM/F12 medium (1:1) (Sigma-Aldrich, Tokyo, Japan) containing human recombinant N2, EGF and bFGF (20 ng/ml; Invitrogen, Carlsbad, CA, USA). The SHG-44 cell line is a differentiated human glioma cell line [16]. The SHG-44 cells were cultured in PRI-1640 supplemented with 10% of fetal bovine serum. Both cell lines were maintained at 37°C in a 5% CO_2_ atmosphere, and were used to study the effects of BNCT.

The cells were collected with trypsin/EDTA during the logarithmic phase, and were seeded in dishes containing nutrient medium. The cells were incubated for 24 h with 5 mM of boronophenylalanine (BPA) (Boron Biologicals Inc., Raleigh, NC, USA)-fructose (Sigma Chemical Co.) complex (50 mg/L of ^10^B equivalent). Medium containing boron was discarded just before the cells were irradiated. BPA-free cells were used as control. The concentration of ^10^B was 1.76 ± 0.28 μg/10^7^ cells in the SU2 cells and 2.50 ± 0.12 μg/10^7^ cells in the SHG-44 cells at the moment of neutron irradiation [17].

### Neutron irradiation

The irradiation experiments were performed at the IHNI-1, using a reactor at the full power of 30 KW with a thermal neutron flux of 1 × 10^9^n/(cm^2^ · s). The components of the reactor neutron source and the irradiation doses calculated according to a previous study [18] are shown in Table 1.

**Table 1:** Total neutron radiation doses according to exposure time

**Group**		^**10**^**B(n,α)**^**7**^**Li (Gy)**	**Other sources (fast neutron, nitrogen neutron, and γ-rays) (Gy)**	**Total dose (Gy)**
SU2	2 min	0.412	0.033	0.445
	4 min	0.824	0.066	0.890
	6 min	1.235	0.100	1.335
	8 min	1.647	0.133	1.780
	10 min	2.059	0.166	2.225
SHG-44	2 min	0.585	0.033	0.618
	4 min	1.170	0.066	1.236
	6 min	1.755	0.100	1.854
	8 min	2.340	0.133	2.472
	10 min	2.925	0.166	3.090

### Clonogenic surviving assay

Cells were seeded in 6-well plates at a density of 200–1000 cells per well after irradiation. Cells were subsequently cultured for 3 weeks and medium was changed every three days. Colonies were stained with 0.1% crystal violet and colonies of more than 50 cells were counted. The percent plating efficiency and surviving fraction were calculated based on the survival of non-irradiated cells.

### Cell proliferation assay

All cells were seeded in 96-well plates at a density of 1000 cells per well after a 4-minute irradiation. After 1, 2 or 3 days of incubation, proliferation of SU2 and SHG-44 cells was assessed using a 3-(4,5-dimethylthiazol-2-yl)-2,5-diphenyl tetrazolium bromide (MTT) assay (Sigma, St Louis, MO, USA). Optical density was recorded using a microplate reader at 495 nm. Cellular proliferation was expressed as a percentage, compared with non-irradiated cells being 100%.

### Cell cycle analysis

Cells were harvested 24 h after a 4-minute irradiation, washed with phosphate buffered solution (PBS), and fixed in dehydrated ethanol overnight at 4°C. After washing, cells were treated with 100 μg RNase A for 30 min at 37°C, and incubated in 10 μg propidium iodide (PI) solution. Cell cycle distribution was determined using a FACScan flow cytometer (Beckman Coulter, Brea, CA, USA).

### Cell apoptosis assay

After BNCT, cells were washed twice in cold PBS and resuspended in cold binding buffer. After incubation with Annexin V-FITC and PI for 15 min, cells were analyzed by a FACScan flow cytometer. The Annexin V FITC-positive cells were early apoptotic cells. Data were expressed as the percentage of Annexin V-FITC-positive cells in the total number of cells counted.

### Western blot

Cell lysates were prepared in RIPA lysis buffer (Cell Signaling Technology, Danvers, MA, USA). Cell debris were removed by centrifugation at 10,000 g for 10 min at 4°C. The protein concentration was determined using the Bradford dye binding assay (Bio-Rad Laboratories, Hercules, CA, USA). Total proteins were fractionated by sodium dodecylsulfonate polyacrylamide gel electrophoresis (SDS-PAGE) at 100 V for 80 min at room temperature, and transferred onto PVDF membrane (Perkin Elmer, Waltham, MA, USA). Target proteins were detected using rabbit monoclonal anti-cyclin-B1, CDK1, cytochrome c and caspase-9 antibodies (Santa Cruz Biotechnology, Santa Cruz, CA, USA) and rabbit polyclonal anti-β-actin antibody (Sigma, St Louis, MO, USA). Immune complexes were detected with horseradish peroxidase (HRP)-conjugated anti-rabbit IgG goat antibodies (Amersham Biosciences, Little Chalfont, UK). The blots were visualized by chemiluminescence (Invitrogen, Carlsbad, CA, USA), and densitometric quantification was analyzed using the Launch Sensi Ansys software.

### Statistical analyses

Each experiment was repeated at least three times. Numerical data are presented as the mean ± standard deviations (SD). Data were analyzed using two-tailed t-test or one-way ANOVA. All statistical analyses were performed using SPSS 10.0 (SPSS Inc., Chicago, IL, USA).

## Results

### Suppression of cell survival by BNCT

**Figure 1:**
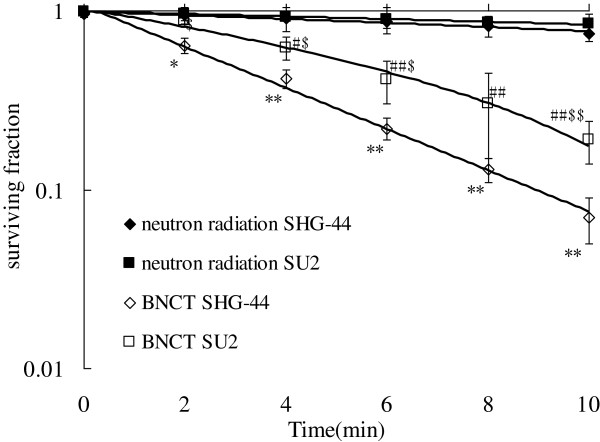
**Surviving curves of SHG-44 and SU2 cells incubated with or without BPA and exposed to 0, 2, 4, 6, 8 and 10 min neutron radiations, determined by clonogenic assay.** *P < 0.05, **P < 0.01 SHG-44 cells after BNCT (◇) vs. neutron radiations alone in SHG-44 cells (◆) for the same time point; ^#^*P* < 0.05, ^##^*P* < 0.01 SU2 cells after BNCT (□) vs. neutron radiations alone in SU2 cells (■) for the same time point; ^$^P < 0.05, ^$$^*P* < 0.01 SU2 cells after BNCT vs. SHG-44 cells after BNCT for the same time point.

After exposure to neutron radiations, the surviving fractions of SU2 and SHG-44 cells were examined using a clonogenic assay. Cell surviving curves were obtained after neutron irradiation according to the linear quadratic model (Figure 1). In both cell lines, surviving ratios were decreased in a dose-dependent manner, but SHG-44 cells were suppressed more strongly than SU2 cells. The surviving fractions of both cells lines pretreated with BPA at the same amount of radiations were significantly decreased compared with BPA-free cells (*P* < 0.05 for SU2 cells when irradiated for 4 min, and *P* < 0.05 for SHG-44 cells when irradiated for 2 min). These results suggest that the survival of SU2 was higher compared with SHG-44 cells using the same BPA pretreatment dose and neutron treatment (*P* < 0.05 for 2, 4, 6 min, and *P* < 0.01 for 10 min).

### Suppression of cell proliferation by BNCT

**Figure 2:**
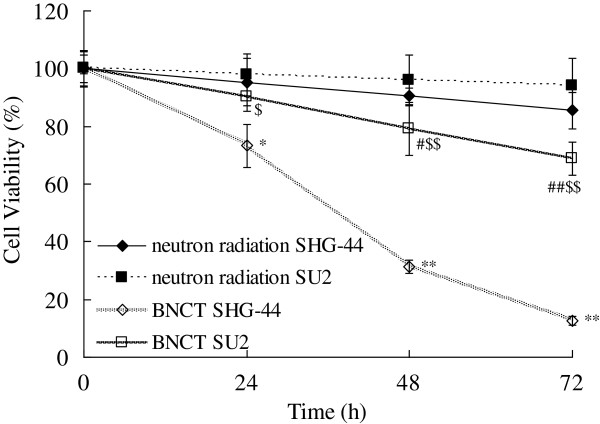
**BNCT inhibited the proliferation of SHG-44 and SU2 cells at 24, 48 and 72 h after a 4-minute neutron irradiation.** The cell viability after neutron radiations alone was used as a control. *P < 0.05, **P < 0.01 compared with neutron radiations alone in SHG-44 cells; ^#^*P* < 0.05, ^##^*P* < 0.01 compared with neutron radiations alone in SU2 cells; ^$^*P* < 0.05, ^$$^*P* < 0.01 compared with BNCT in SHG-44 cells.

We examined the effects of BNCT on the proliferation of SU2 and SHG-44 cells in vitro using a MTT assay. Cell viability after a 4-minute neutron irradiation was assessed daily for 3 days (Figure 2). Treatment with BPA before BCNT inhibited proliferation of the two cell lines in a time-dependent manner. A 32% inhibition of SU2 growth (*P* < 0.01) and 88% in SHG-44 (*P* < 0.01) were observed 72 h after BNCT. SU2 cells viability was significantly decreased 48 h (*P* < 0.05) after BNCT, and this decrease occurred at 24 h in SHG-44 cells (*P* < 0.05). These results suggest that the proliferation of SU2 GSPC was decreased after BNCT.

### Induction of cell cycle arrest by BNCT

**Figure 3:**
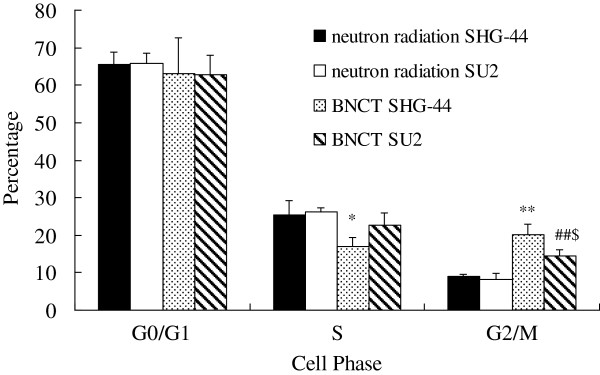
**Cell cycle distribution of SHG-44 and SU2 cells after a 4-minute neutron irradiation.** *P < 0.05, **P < 0.01 compared with neutron radiations alone in SHG-44 cells; ^##^*P* < 0.01 compared with neutron radiations alone in SU2 cells; ^$^*P* < 0.05 compared with BNCT in SHG-44 cells.

Cell cycle arrest in SU2 and SHG-44 cells was investigated by flow cytometry after neutron irradiation (Figure 3). After BNCT, there was no increase of G0/G1 phase cells. The proportion of SHG-44 cells in the G2/M phase was increased from 9.12 ± 0.52% to 20.13 ± 2.85% (*P* < 0.01). However, the proportion of cells in the S phase was decreased from 25.40 ± 3.86% to 16.85 ± 2.52% (*P* < 0.05). In SU2 cells, the G2/M population was significantly increased from 8.09 ± 1.67% to 14.60 ± 1.64% (*P* < 0.01) after BNCT, and the S population was decreased from 26.10 ± 1.24% to 22.57 ± 3.42%. These results indicate that cell cycle was arrested at the G2/M checkpoint after BNCT, and that this arrest was more important in SHG-44 cells (*P* < 0.05).

### Induction of apoptosis by BNCT

**Figure 4:**
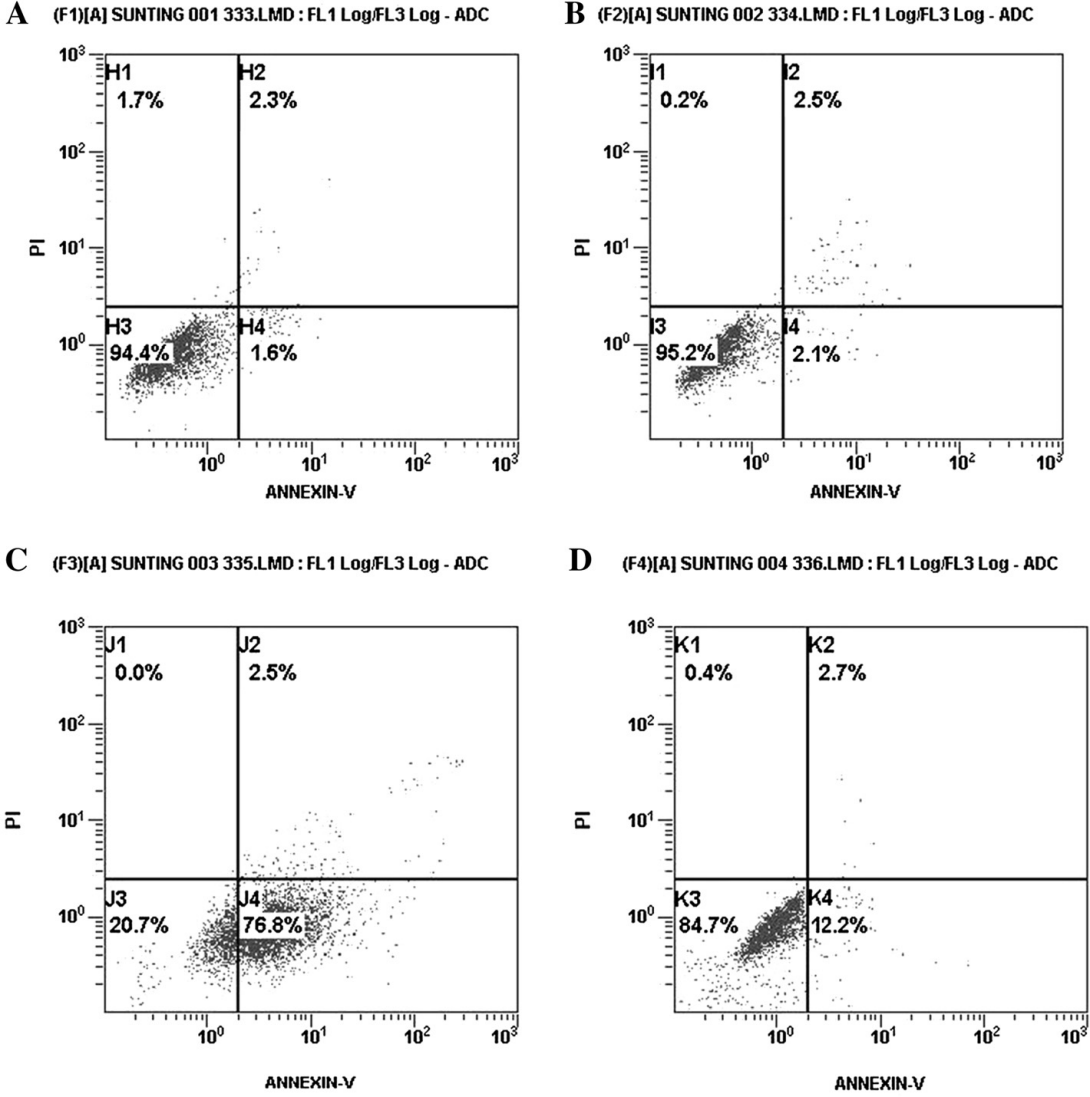
**Proportion of apoptotic SHG-44 and SU2 cells after a 4-minute neutron irradiation.** Apoptotic rate of BNCT in SHG-44 cells **(C)** was significantly increased compared with neutron radiations alone in SHG-44 cells **(A)**, neutron radiations alone in SU2 cells **(B)** and BNCT in SU2 cells **(D)**.

After neutron irradiation, apoptosis of SHG-44 and SU2 cells was quantitatively measured using flow cytometry. As shown in Figure 4, the average apoptotic rate of SHG-44 cells was higher after BNCT (75.2 ± 4.81%) compared with neutron radiations alone (1.9 ± 0.19%) (*P* < 0.01), while the apoptotic proportion in SU2 cells after BNCT was only 11.5 ± 0.87%. These results suggest that the apoptotic ratio of SHG-44 cells was higher compared with SU2 cells (*P* < 0.01) after BNCT using the same dose of BPA and neutron radiations.

### Effects of BNCT on cyclin B1, CDK1, caspase-9 expression and release of cytochrome c

**Figure 5:**
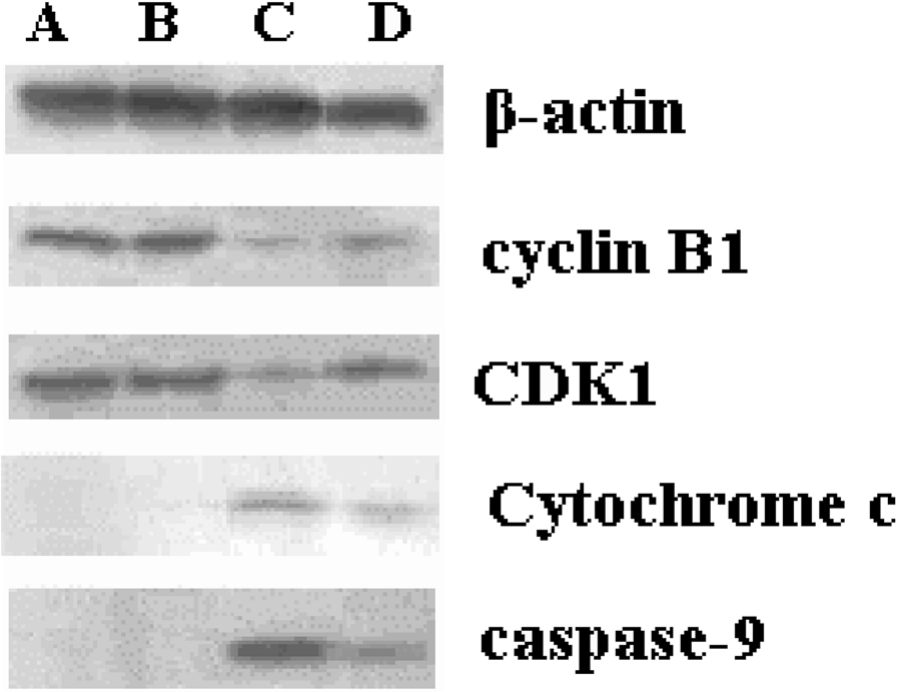
**Protein expression associated with cell cycle and apoptosis.** After a 4-minute irradiation, protein expression was changed in neutron radiations alone in SHG-44 cells **(A)**, neutron radiations alone in SU2 cells **(B)**, BNCT in SHG-44 cells **(C)** and BNCT in SU2 cells **(D)**.

As G2/M phase-associated proteins, cyclin B1 and CDK1 were determined in SHG-44 and SU2 cells after neutron irradiation. As shown in Figure 5, a more obvious decrease in cyclin B1 and CDK1 levels were observed in SHG-44 cells compared with SU2 cells after BNCT. Cytochrome c levels in cytosol, a marker of apoptosis, were increased in SHG-44 and SU2 cells treated with BNCT, and more obvious increases were observed in SHG-44 compared with SU2 cells. Consistently, cleavage of caspase-9, an additional marker of apoptosis, was also increased in both cell lines following BNCT. The relative levels of target proteins expression were normalized against protein levels of β-actin assessed in the same experiments.

## Discussion

The present study confirmed that GSPC could be killed by BNCT, although higher radiation doses were needed compared with differentiated glioma cells. A previous study showed that CD133+ cells were more resistant to ionizing radiations than the corresponding CD133− cells [19]. SU2 GSPC, compared with differentiated glioma SHG-44 cells, has been confirmed to be radioresistant to conventional X-rays radiotherapy, while survival was decreased in SHG-44 cells with increasing doses of X-rays [12]. The same trend was observed in SU2 cells, but their survival was significantly higher than SHG-44 cells using identical X-ray doses, and the ratio of CD133+ cells was increased 3.0-3.6 times compared with before irradiation [12]. Utsumi et al. confirmed that X-ray-sensitive P-39 and X-ray-resistant G-361 human melanoma cell lines had similar sensitivity to thermal neutron irradiation after pretreatment with BPA [20]. To date, many studies performed on different cell types showed that cellular viability was decreased after BNCT [20-23]. However, most of the studies on BNCT focused on differentiated cancer cells, and there is no doubt that these cells could be effectively damaged in vitro and in vivo. In this present study, we used a GSPC line (SU2) and a differentiated glioma cell line (SHG-44) to investigate the effects of BNCT on different sub-population of tumor cells. Our previous study demonstrated that the concentration of ^10^B in GSPC SU2 cultured in 5 mM BPA could meet the concentration required for BNCT, although this concentration was lower than in SHG-44 cells [17]. Differences in ^10^B uptake between SHG-44 and SU2 cells might be due to the differences in the metabolism of GSPC compared with differentiated tumor cells. Thus, the total radiation doses were different between SU2 and SHG-44 cells using the same neutron flux. The in vitro effects of BNCT were evaluated comparatively according to ^10^B-content in the cells and the subsequent dosimetry to predict the cell surviving probability [24]. After BNCT, viability was significantly decreased in SU2 cells compared with SHG-44 cells using the same neutron flux, which demonstrated that GSPC were more resistant to BNCT than differentiated glioma cell. Beside the ^10^B(n,α)^7^Li reaction, other effects (including reactions of hydrogen and nitrogen, and γ-rays) are produced by the IHNI-1, decreasing the surviving fraction and viability of BPA-free cells after irradiation by the IHNI-1.

BNCT has also been shown to interfere with cell cycle progression as a function of BPA concentration and thermal neutron dose. High LET radiations, such as α particles, induce more extensive delays in the S or G2 phase compared with low LET radiations [25], due to the different radiobiological effects of high LET particle beams with respect to impacts on cell cycle control [26]. Our results showed that cell cycle was arrested at the G2/M checkpoint in SHG-44 and SU2 cells after BNCT. Cyclin B1 accumulates and forms a kinase complex with CDK1 during the G2 phase, and G2/M arrest has been associated with an inhibition or a delay in the activation of CDK1 in association with their specific regulatory cyclin B1 proteins [27,28]. Thus, we analyzed the levels of cyclin B1 and CDK1 expression, and the results showed a decreased expression of cyclin B1 and CDK1 proteins after BNCT. Faiao-Flores et al. [21] also showed that BNCT treatment reduced the cells in G0/G1 and G2/M phases, which was proven by the reduction of cyclin D1, and increased amounts of fragmented DNA in melanoma cells. Perona et al. [22] showed that cell cycle was arrested in G2/M 24 and 48 h after BNCT, and that cell death was mostly produced by cell necrosis. The p53 protein was up-regulated 24 h after BNCT, which did not seem to be correlated with the p53-dependent G1/S cell cycle arrest. These previous studies concur with our conclusion on G2/M phase arrest induced by BNCT. Meanwhile, cells in the G2/M phase are the most sensitive to ionizing radiations. Exposure of cells to X- or γ-rays results in a division delay including G1 arrest, G2 arrest or S phase delay. The G2 arrest is seen in virtually all eukaryotic cells following high- or low-dose irradiation, which is one mechanism of cell damage in conventional radiotherapy [29]. One possible mechanism underlying an intrinsic radioresistant GSC was the higher activation of early DNA damage cell cycle checkpoint proteins [19].

Apoptosis induced by radiation is a degradative and progressive process. The degradative process is initiated in the target nucleus, ultimately resulting in the quantitative conversion of the target genome into small DNA fragments [29]. An increased apoptosis was demonstrated by flow cytometry in BNCT-treated cells compared with non-irradiated cells. Wang et al. confirmed that BNCT-induced apoptosis was mediated by the Bcl-2/Bax pathway in glioma cells [23]. In the present study, Western blot revealed changes in cytochrome c and caspase-9 proteins associated with BNCT-induced apoptosis. The induction of cytochrome c release into cytosol by neutron irradiation suggested that BNCT might induce apoptosis by the mitochondria-mediated pathway. Increased cytochrome c interacts with Apaf-1, forming a complex to activate caspase-9. The activated caspase-9 is cleaved and thereby induces the activation of other caspases, such as caspase-3, which subsequently contribute to apoptotic cell death. Another study [21] evaluated the mechanisms of neutron irradiation and showed that BNCT induced a decrease in mitochondrial potential, causing cell death in B16F10 murine melanoma. DNA fragmentation in B16F10 melanoma was extremely significant, but death of these cells did not occur by means of intrinsic apoptosis, because the increase of phosphorylated caspase-3 did not take place. During conventional X-ray radiotherapy, exposure to ionizing radiations elicits a preferential activation of the DNA damage response pathway, while release of cytochrome c into cytosol and activation of caspase-9 occur after exposure to ionizing radiation [30]. Mitochondrial function may regulate cell radiosensitivity and radiation-induced cell cycle G2 checkpoint activation, including protein changes in cyclin B1 and CDK1 [31]. Radioresistance mechanisms of GSC include a decrease of apoptotic cells ratio and cleaved caspase-9 [19]. Perhaps the reasons that apoptotic percentage of SHG-44 and SU2 cells was significantly different were relative to not only difference of boron uptake but also radiation resistance and injury repair of GSPC. Therefore, arrest in the G2/M phase and induction of mitochondria-associated apoptosis are possible mechanisms of BNCT on proliferative inhibition in SHG-44 and SU2 cells.

A previous study suggested that CD133+ GSC were radioresistant due, in part, to enhanced DNA repair [19]. Radiation-induced delays in the G1, S and G2 phases of the cell cycle are thought to allow periods during which cells survey and repair DNA damage. Cells will promote the apoptotic pathway if DNA damage cannot be repaired. Following exposure to high LET radiations, the clustering of lesions in DNA induced by ionizing radiation is thought to play an important role in long-term biological effects. Thus, BNCT would offer an appreciable therapeutic advantage over X-ray or γ-ray therapy.

Some clinical results demonstrated that the median surviving times of GBM patients who received a single BNCT treatment with BPA or BPA and BSH, or BNCT combined with conventional radiotherapy using epithermal neutron sources from Finnish, Swedish, or Japanese reactors, were equivalent or superior to those receiving only conventional radiation therapy. These results might be due to GSPC damage in the presence of sufficient amounts of boron. The median surviving times of GBM patients who received BNCT with thermal and epithermal from Finnish Fir-1, Swedish R2-o and Japanese JRR-4 or KURR reactors were 11.0-21.9, 17.7 and 23.3-27.1 months, respectively [8]. However, these results were not satisfying enough. Boron uptake, especially in radio- and chemo-resistant GSC, plays a key role in the biological effects of BNCT. Some experimental methods, including targeting molecules overexpressed on the tumor surface, preloading compounds, increasing total dose, extending infusion time, and changing route of administration could increase the boron content in tumor cells, thus improving treatment efficacy. With the improvement of neutron-generating instruments, BNCT could more effectively damage tumor cells, without affecting peripheral normal cells.

## Conclusion

GSPC, which are resistant to current clinical radiotherapy, were effectively killed by BNCT in vitro using a prolonged neutron irradiation, but not damaged by neutron radiation with BPA-free. Radiosensitivity of GSPC was decreased compared with differentiated glioma cells using the same dose of thermal neutron exposure and BPA pretreatment at least due partly to low uptake of boron. BNCT induced GSPC cell cycle arrest in the G2/M phase and apoptosis by the mitochondria-mediated pathway. BNCT can damage GSPC, which are resistant to X- and γ-rays and play a key role in glioma recurrence, as long as the cells receive enough high LET radiations from thermal neutrons. Thus, BNCT could offer an appreciable therapeutic advantage to prevent tumor recurrence, and may become a promising treatment for glioma.

## Abbreviations

BNCT: Boron neutron capture therapy; GSPC: Glioma stem/progenitor cell; BPA: Boronophenylalanine; 10B: Boron-10; IHNI-1: In-hospital neutron irradiator; GSC: Glioma stem cells; GBM: Glioblastoma multiforme; LET: Linear energy transfer; PBS: Phosphate buffered solution.

## Competing interests

The authors declare that they have no competing interests that could inappropriately influence this work.

## Authors’ contributions

TS carried out experiments and performed data acquisition, data analysis and drafting of the manuscript. YxZ contributed significantly to the design of the study. ZzZ performed neutron irradiation and dose calculations. BL performed Western blot assays. GL performed cell cultures. XsX, YxW and JW assisted TS in carrying out experiments. ZwD participated in experimental design and coordination. All authors read and approved the final manuscript.
